# Preoperative hemoglobin levels and mortality outcomes after hip fracture patients

**DOI:** 10.1186/s12893-023-02174-5

**Published:** 2023-09-01

**Authors:** Bassem I. Haddad, Mohammad Hamdan, Mohammad Ali Alshrouf, Abdallah Alzubi, Ahmed Khirsheh, Ahmad Al-Oleimat, Mohammad Aldabaibeh, Rayyan Al-Qaryouti, Waleed Abulubbad, Munther Al-Saber, Mohammad Jabaiti, Abdulrahman M. Karam

**Affiliations:** 1 Department of Special Surgery, Division of Orthopedics, School of Medicine, The University of Jordan, Amman, 11942 Jordan; 2 Medical Internship, Jordan University Hospital, The University of Jordan, Amman, 11942 Jordan; 3 The School of Medicine, The University of Jordan, Amman, 11942 Jordan; 4 Medical Internship, Arab Medical Center, Amman, 11181 Jordan

**Keywords:** Hip fracture, Anemia, Hemoglobin, Renal disease, Preoperative, Elderly, Mortality

## Abstract

**Purpose:**

Hip fracture surgery is associated with a risk of morbidity and mortality, with admission hemoglobin levels being a significant predictor of mortality risk. The aim of this study is to evaluate the relationship between the preoperative hemoglobin (Hb) levels and mortality in patients who underwent hip fracture surgeries, with the goal of enhancing prognosis prediction and reducing complications within this patient subset. In addition, to assess the characteristics of patients at a higher risk of postoperative mortality.

**Methods:**

This retrospective study was conducted at Jordan University Hospital, a single tertiary care and educational center. It included patients with hip fractures who underwent surgical repair at the Department of Orthopedic Surgery and were recruited between December 2019 and February 2022. We examined the relationships between preoperative hemoglobin status and variables such as age at admission, gender, fracture type, surgery type, comorbidities, duration of hospital stay, intensive care unit (ICU) admission, and survival outcomes.

**Results:**

We included 626 patients; the mean age was 76.27 ± 9.57 years. 3-month and 6-month mortality rates were 11.2% and 14.1%, respectively. The highest mortality was observed in patients aged over 80 years (n = 53/245, 21.6%), and in male patients (n = 53/300, 17.7%). The Hb level upon admission was lower in individuals who died within 6 months compared to those who survived (10.97 ± 2.02 vs. 11.99 ± 2.39, p < 0.001). In multivariate analysis, the independent factors that were statistically significant in the model included gender (OR = 1.867; 95% CI 1.122–3.107, p = 0.016), age (OR = 1.060; 95% CI 1.029–1.092; p < 0.001), hemoglobin level upon admission (OR = 0.827; 95% CI 0.721–0.949; p = 0.007), history of renal disease (OR = 1.958; 95% CI 1.014–3.784; p = 0.045), length of hospital stay (OR = 1.080; 95% CI 1.036–1.126; p < 0.001), and ICU admission (OR = 1.848; 95% CI 1.049–3.257; p = 0.034).

**Conclusion:**

Our study illustrates that low hemoglobin levels, history of renal disease, along with male gender, advanced age, extended hospital stays, and ICU admission were significantly associated with 6-month mortality. Future investigations should consider assessing varying degrees of anemia based on hemoglobin concentrations to provide a more comprehensive understanding of anemia’s impact on mortality.

**Mini-abstract:**

This study investigated the relationship between preoperative hemoglobin levels, patient characteristics, and mortality in patients who underwent hip fracture surgeries. The results showed that lower hemoglobin levels, history of renal disease, male gender, advanced age, extended hospital stays, and ICU admission were significant predictors for mortality.

**Supplementary Information:**

The online version contains supplementary material available at 10.1186/s12893-023-02174-5.

## Introduction

In recent years, the increasing life expectancy and growing proportion of elderly individuals in the population have made hip fractures a major global public health concern [1]. These events often lead to physical disability, difficulties with daily activities, and higher mortality rates [2, 3]. Furthermore, the financial burdens of hip fractures cannot be overlooked with far-reaching effects on economics and society at large and is estimated to reach 4.5 million by the year 2050 [4, 5]. Due to factors such as decreased bone density, multiple medications for comorbidities, a higher risk of falls due to visual impairment, and movement difficulties, elderly individuals, particularly females, are at an increased risk for hip fractures [6, 7]. Various surgical techniques, including dynamic hip screw (DHS), partial hip replacement (PHR), and intramedullary nail (IMN), are commonly used to treat hip fractures.

Anemia is a common problem among the elderly and is associated with diminished functional status as well as higher rates of mortality and morbidity [8]. Although many cases in the elderly have unknown etiologies, chronic illnesses, iron, vitamin B12, and folate deficiencies, malignancies, and gastrointestinal bleeding are among the most common causes of anemia [9]. Several studies tried to assess the relationship between preoperative hemoglobin levels and mortality rates in patients with hip fractures. Some of these studies found that preoperative anemia, defined as hemoglobin levels below 12 g/dL, is an independent risk factor for postoperative mortality and complications in hip fracture patients, where patients with higher hemoglobin levels on admission had better outcomes, shorter hospital stay, lower rates of readmission and lower mortality rates [10–12]. However, other studies have reached different conclusions; Vochteloo et al. found that preoperative anemia had no effect on the length of hospital stay or postoperative mortality [6]. Furthermore, Hagino et al. found that anemia was related to age, fracture type, and ambulatory status, but not in-hospital mortality [13].

Although some studies have suggested a link between preoperative anemia and an increased risk of postoperative complications and poor outcomes in hip fracture patients [14, 15], the relationship between preoperative hemoglobin levels and surgical outcomes is not yet fully understood. Therefore, the primary objective of this study is to investigate the impact of preoperative anemia on mortality rates after hip fracture surgery. Furthermore, to identify the characteristics of patients, including demographics and pertinent clinical data, who are at a higher risk of postoperative mortality. The findings of this study will help us to better predict the prognosis of hip fracture patients by improving our understanding of the relationship between preoperative hemoglobin levels, other patients’ characteristics and surgical outcomes. We hypothesize that the preoperative hemoglobin level and patient characteristics increase the risk and can be used to predict postoperative mortality rates in patients with hip fractures.

## Methodology

### Study design

This retrospective study was carried out over a period of 2 years and 3 months from December 2019 to February 2022 at Jordan University Hospital (JUH), a tertiary care and educational center on patients who suffered from hip fractures and underwent hip fracture surgeries. The study was conducted in accordance with the ethical regulations of the Declaration of Helsinki, and the study protocol was approved by the institutional review board (IRB) at Jordan University Hospital (reference number 10/2022/5663; 1 March 2022). This study is presented in line with STROBE checklist (STROBE checklist available in the Supplementary materials).

The target population of this study consists of patients who had a femur neck fracture, an intertrochanteric fracture or a subtrochanteric fracture, performed their surgery at JUH, and had their preoperative and postoperative notes available in the JUH database. Exclusion criteria included patients who had their surgery outside of JUH or who were lost to follow-up before 6 months. Therefore, a total of 632 patient records were collected, out of which 626 patients who met the inclusion criteria were included in this study.

### Data collection

Data collection was performed by retrospectively reviewing the electronic medical records of the patients. Information obtained included demographics such as age upon admission, gender and relevant clinical data, including comorbidities, length of hospital stay, intensive care unit (ICU) admission, type of fracture (Femur Neck, Intertrochanteric, or Subtrochanteric), type of surgery (Partial Hip Replacement, Intramedullary Nail, or Dynamic Hip Screw), and a hematological panel (Hb level and RDW) upon admission.

Based on our laboratory reference range used in JUH, lower cut-off limit for hemoglobin was 12 g/dL, while for red cell distribution width (RDW) it was 15%. The relationship between both parameters on admission was investigated with regard to overall mortality.

### Outcome measurement

Post-operative mortality was assessed by following up with patients at 3 and 6 months after surgery. In addition, patient characteristics were assessed to identify the patient at higher risk of postoperative mortality. These follow-up data were obtained from patients themselves or their families, either through clinic visits or phone calls.

### Statistical analysis

All data were entered into Microsoft Excel software, where they were recorded, cleaned and polished. The data were described using variability analysis in the form of means (standard deviation). The sociodemographic factors were calculated and provided as frequencies (percentages) using standard descriptive statistical parameters. Pearson’s chi-square test, Fisher’s exact test, and Independent-samples t-test were used to assess significant differences among the different groups of patients. Logistic regression, represented as an odds ratio (OR) 95% confidence interval (CI), was performed to assess the impact of several factors on the likelihood of mortality in patients undergoing hip fracture surgery. Youden’s index was used to estimate the preoperative level of hemoglobin threshold to predict the mortality outcome. The receiver operating characteristic curve was used to test the sensitivity and specificity of the value. SPSS version 25.0 (Chicago, USA) was used for the analysis. All variables with a p < 0.05 were considered statistically significant.

## Results

A total of 626 patients who underwent surgery for hip fractures between December 2019 and February 2022 were included in the study. Of these, 326 (52.1%) were female, the mean age was 76.27 ± 9.57 years, and 511 (81.6%) were over 70 years old. The most common type of fracture was intertrochanteric (60.5%), followed by femoral neck (34.4%) and subtrochanteric (5.1%) fractures. The most frequently performed surgery (41.7%) was intramedullary nail, followed by partial hip replacement (33.9%), and dynamic hip screw (24.4%). The mean hemoglobin level upon admission was 11.85 ± 2.35 g/dL, and the mean RDW was 15.53 ± 1.99%. Age groups, the type of surgery, the type of fracture, comorbidities, length of hospital stay, and ICU admission are summarized in Table 1.

**Table 1:** Sociodemographic characteristics and differences between groups

Characteristics		Value (n = 626)
Gender	Male	300 (47.9%)
	Female	326 (52.1%)
Age upon admission, mean ± SD		76.27 ± 9.57
Age groups	< 60 years	34 (5.4%)
	60–69 years	81 (12.9%)
	70–79 years	266 (42.5%)
	> 80 years	245 (39.1%)
Type of surgery	Partial hip replacement	212 (33.9%)
	Intramedullary nail	261 (41.7%)
	Dynamic hip screw	153 (24.4%)
Type of fracture	Femur neck fracture	215 (34.4%)
	Intertrochanteric fracture	378 (60.5%)
	Subtrochanteric fracture	32 (5.1%)
Comorbidities	Hypertension	426 (68.1%)
	Diabetes mellitus	309 (49.4%)
	Cardiovascular diseases	183 (29.2%)
	Cerebrovascular accident	76 (12.1%)
	Renal diseases	86 (13.7%)
	Malignancy	44 (7.0%)
	Pulmonary diseases	37 (5.9%)
	Dementia	37 (5.9%)
	Neurological disease	18 (2.9%)
Length of hospital stay, mean ± SD		8.21 ± 5.60
ICU admission	Yes	166 (26.5%)
	No	460 (73.5%)
Hemoglobin level at admission, mean ± SD		11.85 ± 2.35
Hemoglobin upon admission (g/dl)	< 12	347 (55.4%)
	≥ 12	279 (44.6%)
RDW upon admission, mean ± SD		15.53 ± 1.99
RDW upon admission	< 15	287 (45.8%)
	≥ 15	339 (54.2%)

Note: SD, standard deviation; RDW, red cell distribution width

Anemia (defined as hemoglobin < 12.0 g/dL) was observed in 347 (55.4%) patients. Patients who died within six months were more likely to have anemia than those who survived (69.3% vs. 53.2%, respectively) (p = 0.003). Table 2 represents the details of the characteristics of 6-month mortality after hip surgery.

**Table 2:** 6-month mortality after hip surgery

Variables	n (%)
Overall mortality	
In-hospital mortality	30 (4.8%)
3-month mortality	70 (11.2%)
6-month mortality	88 (14.1%)
6-month mortality classified by age	
< 60 years (n = 34)	2 (5.9%)
60–69 years (n = 81)	9 (11.1%)
70–79 years (n = 266)	24 (9.0%)
> 80 years (n = 245)	53 (21.6%)
6-month mortality classified by sex	
Male (n = 300)	53 (17.7%)
Female (n = 326)	35 (10.7%)
6-month mortality classified by surgery type	
Intramedullary nail (n = 261)	41 (15.7%)
Partial hip replacement (n = 212)	27 (12.7%)
Dynamic hip screw (n = 153)	20 (13.1%)
6-month mortality classified by fracture type	
Femur neck fracture (n = 215)	27 (12.6%)
Intertrochanteric fracture (n = 378)	59 (15.6%)
Subtrochanteric fracture (n = 32)	2 (6.3%)
6-month mortality classified by health status	
Medically free patients (n = 72)	6 (8.3%)
Comorbid patients (n = 554)	82 (14.8%)

During the follow-up period, 88 (14.1%) died, with 30 deaths occurring in the hospital, 40 deaths within 3 months, and 18 deaths within 6 months after the surgery. Most of the deaths were observed in patients with age greater than 80 years (60.2%) and most were in male patients (60.2%). Upon comparing the characteristics of patients who died within six months of admission (10.97 ± 2.02) and those who survived (11.99 ± 2.39), patients who had died exhibited lower Hb level at admission (p < 0.001). In addition, there was a significant mean difference in mortality rate between patients with RDW levels (p = 0.033). There were no significant differences in type of fracture, or type of surgery between patients who died and those who survived. Table 3 compares the sociodemographic, hemoglobin, red cell distribution width (RDW), comorbidities, length of hospital stay, ICU admission, and survival between the survivors and the deceased.

**Table 3:** Hemoglobin, red cell distribution width (RDW), Comorbidities, and Survival

6-month Mortality		Survivors (n = 538)	Deceased (n = 88)	*p*-Value
Age, mean ± SD		75.60 ± 9.30	80.40 ± 10.15	**< 0.001** ^†^
Age groups	< 60 years	32 (5.9%)	2 (2.3%)	
	60–69 years	72 (13.4%)	9 (10.2%)	
	70–79 years	242 (45.0%)	24 (27.3%)	**< 0.001** ^‡‡^
	> 80 years	192 (35.7%)	53 (60.2%)	
Gender	Male	247 (45.9%)	53 (60.2%)	**0.009** ^‡^
	Female	291 (54.1%)	35 (39.8%)	
Hemoglobin level upon admission, mean ± SD		11.99 ± 2.39	10.97 ± 2.02	**< 0.001** ^†^
RDW level upon admission, mean ± SD		15.46 ± 1.99	15.95 ± 1.97	0.**033**^†^
Hemoglobin < 12 g/dl		286 (53.2%)	61 (69.3%)	**0.003** ^‡^
RDW ≥ 15		280 (52%)	59 (67.0%)	**0.011** ^‡^
Health status	Medically free	66 (12.3%)	6 (6.8%)	0.091^‡^
	Comorbid	472 (87.7%)	82 (93.2%)	
Comorbidities	Hypertension (n = 426)	369 (68.6%)	57 (64.8%)	0.276^‡^
	Diabetes (n = 309)	261 (48.5%)	48 (54.5%)	0.175^‡^
	Cardiovascular diseases (n = 183)	151 (28.1%)	32 (36.4%)	0.074^‡^
	Cerebrovascular accidents (n = 76)	64 (11.9%)	12 (13.6%)	0.376^‡^
	Renal disease (n = 86)	68 (12.6%)	18 (20.5%)	0.**040**^‡^
	Malignancy (n = 44)	36 (6.7%)	8 (9.10%)	0.267^‡^
	Pulmonary diseases (n = 37)	29 (5.4%)	8 (9.1%)	0.133^‡^
	Dementia (n = 37)	30 (5.6%)	7 (8.0%)	0.247^‡^
	Neurological diseases (n = 18)	15 (2.8%)	3 (3.4%)	0.476^‡^
Length of hospital stay, mean ± SD		7.66 ± 4.67	11.64 ± 8.82	< 0.001^†^
ICU admission	Yes	415 (77.1%)	45 (51.1%)	< 0.001^‡^
	No	123 (22.9%)	43 (48.9%)	
Type of fracture	Intertrochanteric	319 (59.4%)	59 (67.0%)	
	Femur neck	188 (35.0%)	27 (30.7%)	0.251^‡‡^
	Subtrochanteric	30 (5.6%)	2 (2.3%)	
Type of surgery	Intramedullary nail	220 (40.9%)	41 (46.6%)	
	Partial hip replacement	185 (34.4%)	27 (30.7%)	0.601^‡‡^
	Dynamic hip screw	133 (24.7%)	20 (22.7%)	

^*†*^_*Independent−samples t−test*_^*‡*^_*Fisher’s exact test*_^*‡‡*^_*Chi−square test for independence*_

Logistic regression was performed to assess the impact of a number of factors on the likelihood that patients undergoing hip fracture surgery mortality risk. Variables with a p value < 0.1 in univariate analysis were entered into the model. The model contained the following independent variables, gender, age, hemoglobin level upon admission, RDW level upon admission, renal disease, length of hospital stay, and ICU admission. The full model containing all predictors was statistically significant, p < 0.001, indicating that the model was able to distinguish between respondents who most likely would die after hip fracture surgery. The model as a whole explained between 11.4% (Cox and Snell R square) and 20.7% (Nagelkerke R squared), and correctly classified 87.0% of cases. As shown in Table 4, the independent variables made a unique statistically significant contribution to the model gender (OR = 1.867; 95% CI 1.122–3.107, p = 0.016), age (OR = 1.060; 95% CI 1.029–1.092; p < 0.001), hemoglobin level upon admission (OR = 0.827; 95% CI 0.721–0.949; p = 0.007), history of renal disease (OR = 1.958; 95% CI 1.014–3.784; p = 0.045), length of hospital stay (OR = 1.080; 95% CI 1.036–1.126; p < 0.001), and ICU admission (OR = 1.848; 95% CI 1.049–3.257; p = 0.034). Moreover, the hemoglobin level upon admission of 10.45 g/dL, derived from Youden’s index, was found to be the threshold for determining mortality with a sensitivity of 0.466 and a specificity of 0.757.

**Table 4:** Analysis of predictors of 6-month mortality after hip fracture surgery

	*p*-Value	Odds Ratio (OR)	Lower 95% CI for OR	Upper 95% CI for OR
Age upon admission	< 0.001	1.060	1.029	1.092
Gender (Female = Reference)	0.016	1.867	1.122	3.107
Hemoglobin level upon admission	0.007	0.827	0.721	0.949
RDW level upon admission	0.825	1.014	0.895	1.1.49
Renal Diseases (No = Reference)	0.045	1.958	1.014	3.784
Length of hospital stay	< 0.001	1.080	1.036	1.126
ICU admission (No = Reference)	0.034	1.848	1.049	3.257

## Discussion

The aim of this retrospective study was to assess the relationship between preoperative hemoglobin levels, patient’s characteristics, and postoperative mortality in a sample of 626 patients who underwent hip fracture surgeries. Furthermore, this study aimed to explore additional factors that could potentially impact postoperative outcomes. Our univariate analysis demonstrated that low levels of hemoglobin upon admission, level of RDW upon admission, with a range of patient attributes including age, gender, renal disease, length of hospital stay, and ICU admission were associated with 6-month mortality. These variables were included in the logistic regression model in order to control for possible confounding effect. Which showed that preoperative low hemoglobin, history of renal disease, increased age, male gender, length of hospital stay, and ICU admission were significant predictors associated with 6-month mortality.

Anemia is highly prevalent in patients with hip fractures, with 12.3% having Hb levels less than 10 g/dL and 40.4% having Hb levels less than 12 g/dL [10, 16]. The cut-off Hb level, which has been linked to high mortality after hip fracture, is debatable. According to the World Health Organization (WHO), anemia is defined as having Hemoglobin (Hb) levels lower than 12 g/dL in females and lower than 13 g/dL in males [17]. Several studies used a cut-off of less than 10 g/dL to determine preoperative low Hb level at admission [18, 19], whereas other studies used a threshold of less than 12 g/dL, as we did, without considering gender differences [20]. Our findings corroborated the previously published studies addressing the link between anemia and mortality in patients with hip fracture. The literature demonstrated that anemia increases the risk of 90-day and 180-day mortality amongst hip fracture patients [21–24]. In a large study conducted by Kovar et al., the authors used the WHO definition of anemia with respect to sex differences. The study identified an association between Hb levels at admission and short-term mortality [23]. Moreover, anemia may be corrected by transfusion prior to hip fracture surgery, however there is presently no consensus on who should get a transfusion or when, and no established threshold values are used to define preoperative anemia [25]. A recent research found that preoperative transfusions to elevate hemoglobin to more than 10 g/dL reduced mortality risk by 50% in osteoporotic hip fracture patients [26]. In our study, the hemoglobin level upon admission of 10.45 g/dL, derived from Youden’s index, was found to be the threshold for determining mortality with a sensitivity of 0.466 and a specificity of 0.757.

Furthermore, the relationship between age and 6 month mortality was statistically significant as the mean age of patients alive at 6 months post-operatively was 75.60 years while the mean age of those who died within 6 months after surgery was 80.40 years. These results are in line with other studies which showed that higher age is related to an increased risk of mortality after hip fractures [27–30]. A previous study conducted in Sweden found that age played a significant role in the increased relative risk for mortality at 4 and 12 months following hip fractures [27]. Furthermore, higher mortality rates were observed in patients with an age greater than 80 years, which goes hand in hand with our findings [31]. The observed relationship between age and mortality among hip fracture patients may be attributed to a multifactorial effect and it was reported that individuals aged 80 years and above have 4.3 times higher odds of not regaining basic mobility, which could explain our findings [32]. In contrast to our findings, a study conducted in Germany showed that advanced age was not an independent risk for in-hospital mortality following a hip fracture [33]. This highlights the importance of additional research to fully understand this subject.

Based on our results, there was no statistically significant difference in the 6-month mortality when comparing the different types of fractures. However, other studies have showed that intertrochanteric fractures have been linked to a higher risk of morbidity and mortality when compared to other types of hip fractures [34]. Even when accounting for age and comorbid conditions by using multivariable analyses, several reports found significantly higher mortality rates among patients with an intertrochanteric fracture [35, 36]. Nevertheless, in a retrospective study of 2,674 patients, no significant differences in mortality were reported between patients with femoral neck and pertrochanteric hip fractures during an average follow-up of 2.6 years [37]. These findings are consistent with the results obtained by Panula et al., which found no difference in mortality or cause of death between fracture types [38]. These contradictory findings do point to the fact that differences in fracture patterns may not be universal, and, hence, there is a need to conduct further large-scale prospective studies to evaluate the association and differences in postoperative mortality.

Our sample consisted of 300 males and 326 females, demonstrating a nearly equal distribution of gender. Our study found no significant differences in in-hospital mortality rates between male and female patients. However, gender was found to be a significant factor in 6-month mortality, as males had a higher mortality rate (60.2%) compared to females (39.8%). Our findings were consistent with the results of studies done in Sweden and Denmark, which stated that male gender was found to be associated with an increase in the risk of mortality after hip fracture events [27, 28]. Other studies corroborate our findings that male gender is a risk factor for mortality within 3 and 6 months after hip fracture after adjustment for comorbidities among the patients, the mobility of the patients before the fracture, age, and the fracture type [39, 40].

Despite all the literature that studied the relationship between the patient’s gender and mortality, a strong explanation for the differences among genders in mortality after hip fracture events has not yet been stated [39]. One explanation was that male health state was less stable at the time of the fracture, increasing vulnerability to post-fracture infections such as pneumonia and septicemia, and therefore increased mortality [41].

Our study results failed to demonstrate a significant association between mortality rates following hip fracture surgeries and cardiovascular diseases among our patients. However, several studies showed that a history of preexisting cardiovascular diseases, including ischemic cardiac disease, acute perioperative myocardial injury and heart failure, was associated with higher in-hospital, 30-day, and 90-day mortality rates [42–44]. When taking into account preoperative hemoglobin levels in patients with cardiovascular diseases, it was found that the risk of adverse outcomes associated with low hematocrit levels was more pronounced in patients with a history of cardiovascular disease [45]. This is an important finding that can help us in understanding the role of cardiovascular comorbidities in predicting the mortality of hip fracture patients. Several large studies in the broader literature reported difficulties in interpreting the complex relationship between anemia and cardiovascular diseases. For instance, Sabatine et al. conducted a study on 39,992 patients who had acute coronary syndrome and found that anemia was a significant predictor of higher 30-day mortality [46].

Hip fractures in chronic kidney disease patients typically result due to malnutrition, diminished muscular strength, and decreased bone density, due to vitamin D and parathyroid hormone abnormalities [47]. Renal diseases were found to have a significant association with the 6-months mortality rate in patients with hip fractures, according to the results of our univariate analysis. As our sample had 86 patients with renal diseases, 18 patients of whom have died after 6 months of the hip fracture. Moreover, logistic regression analysis showed that renal diseases were a significant predictor of mortality 6 months after hip fracture surgery, which was the strongest risk factor with an odds ratio of 1.958. Our findings are consistent with the existing literature, which suggests that renal diseases may independently contribute to patient mortality following hip fractures [28, 48, 49].

Based on our results, length of hospital stay was considered an independent risk factor for mortality after hip fracture events. These findings are consistent with those of Nikkel et al., who found that shorter hospital stay was associated with lower mortality rates [50]. However, opposite findings have also been reported [51–53]. These contradictory findings could be attributed to variations in the approaches to hip fracture management among different health care systems [50]. Furthermore, ICU admission was recognized as one of the independent predictors of mortality following hip fracture surgery. This finding is supported by the fact that ICU admission is recommended for high-risk patients or those undergoing complex or emergency surgery [54]. Moreover, Kim et al. analyzed the factors influencing admission to the ICU and found that the length between injury and operation, as well as preoperative neurological comorbidities and frequent intraoperative hypotension, were all significant [55].

The findings of this study hold significance in comprehending the varying forms of care that ought to be provided to elderly patients who undergo hip fracture surgeries. However, there are various limitations to this study that should be noted when interpreting the findings, including its retrospective design and single-center setting, thus sampling bias could not be ruled out. In addition to that, Jordan does not have an effective postoperative follow-up system as well as a national computerized medical record database that connects multiple healthcare facilities, therefore our study could be prone to underreporting and loss of follow-up. To address these concerns, further prospective studies are needed in the Jordanian population.

## Conclusion

Based on our retrospective study of patients who underwent surgery for hip fractures, we found that low hemoglobin levels, history of renal disease, male gender, age, length of hospital stay, and ICU admission were independent factors associated with increased 6-month mortality. Our study provides valuable insight into the impact of anemia on mortality after hip fracture surgery, which can help inform clinical decision-making and patient management. In addition, patients with history of renal disease, male gender, advanced age, extended hospital stays, and ICU admission were a predictors for mortality after hip fracture surgery; however, further studies should be conducted on a larger sample size so that the findings may be extrapolated on the Arab region and Middle East region as a whole. We recommend identifying anemia cases in presurgical periods and starting treatment to avoid surgery complications and improve patient outcomes. Furthermore, it is advisable to institute specific guidelines for blood management in hip fracture surgeries across all healthcare institutions. Moreover, further studies are needed to investigate the use of red blood cell transfusions before, during, and after hip fracture surgery and establish a clear cutoff for transfusion. Overall, our study highlights the importance of preoperative anemia screening and management in hip fracture patients to improve their outcomes and reduce mortality.

### Electronic supplementary material

Below is the link to the electronic supplementary material.

Supplementary Material 1

## Data Availability

The data from the present research that were utilized and analyzed are available upon request from the corresponding author.
